# COE Inhibits Vasculogenic Mimicry by Targeting EphA2 in Hepatocellular Carcinoma, a Research Based on Proteomics Analysis

**DOI:** 10.3389/fphar.2021.619732

**Published:** 2021-03-31

**Authors:** Zewen Chu, Xin Shi, Gaoyang Chen, Xuejun He, Yayun Qian, Haibo Wang, Li Tao, Yanqing Liu, Wei Jiang, Jue Chen

**Affiliations:** ^1^ Department of Oncology, The Second People's Hospital of Taizhou Affiliated to Medical College of Yangzhou University, Yangzhou, China; ^2^ The Key of Cancer Prevention and Treatment of Yangzhou University, Yangzhou, China; ^3^ Institution of Integrated Traditional Chinese and Western Medicine, Medical College, Yangzhou University, Yangzhou, China; ^4^ College of Environmental Science and Engineering, Marine Science and Technology Institute, Yangzhou, China; ^5^ Department of Oncology, Affiliated Hospital of Yangzhou University, Yangzhou, China

**Keywords:** vasculogenesis mimicry, hepatocel lular carcinoma, EphA2, protemics, cancer treatment

## Abstract

New strategies and drugs are urgently needed to improve the treatment of hepatocellular carcinoma (HCC). Vasculogenic mimicry (VM) has been elucidated being associated with the progression of HCC and anti-VM could be a promising strategy. *Celastrus orbiculatu* *s* extract (COE), a mixture of 26 compounds isolated from the Chinese Herb *Celastrus Orbiculatus* Vine, has been elucidated to be able to disrupt VM formation in HCC. This study aims to dissect and identify the potential targets of COE on anti-VM formation both *in vitro* and *in vivo* that are distinct from our previous study. Proteomics analysis was used to identify differential proteins in HCC cells treated with or without COE (Data are available *via* ProteomeXchange with identifier PXD022203). Cells invasion was examined using Transwell. Matrigel was used to establish a 3-D culture condition for VM formation *in vitro*. RT-PCR and Western Blot were used to examine changes of mRNA and protein respectively. Clinical resected samples were applied to confirm association between VM formation and identified targets. Subcutaneous xenograft tumor model was established to observe tumor growth and VM formation *in vivo*. PAS-CD34 dual staining was used to detect VM *in vivo*. A total of 194 proteins were identified to be differentially expressed in HCC cells treated with or without COE. In the 93 down-regulated proteins EphA2 stood out to be regulated on both RNA and protein level. Disruption EphA2 using COE or NVP inhibited VM formation and decreased VM associated biomarkers. In xenograft mouse model, COE inhibited tumor growth and VM formation via down-regulating EphA2. Taken together, our results indicate that COE could be used in HCC treatment because of its promising anti-VM effect.

## Introduction

Hepatocellular carcinoma (HCC) is a common cancer of the digestive system. It has a high morbidity and mortality worldwide (Lu et al., 2019). Because HCC has the characteristics of rich-vascularity and high heterogeneity, patients with HCC often suffer poor prognosis and low quality of life (Ferrer-Fabrega and Forner, 2020). Radical resection or liver transplantation is the main treatments for early-stage liver cancer (Mazzaferro et al., 2020). However, only 20% of the patients meet the radical treatment when newly diagnosed (Raoul and Edeline, 2020). The 5 years survival rate of HCC is only about 20% because of its invasiveness and high recurrence rate (Pinato, 2020). Therefore, new strategies and drugs to improve treatment is urgently needed.

Sufficient blood supply is necessary for the growth and metastasis of cancer cells (Kaur et al., 2016). The success of anti-angiogenesis in oncologic practice also confirms this consideration (Tan et al., 2016). However, anti-angiogenic strategy failed to inhibit progression of some solid tumors, including HCC (Lee et al., 2020). Hence, there might be another vascular system involved in the tumor blood supply. In 1999, researchers found and confirmed a new type of blood supply named vasculogenic mimicry (VM), which is formed by tumor cells independently (Maniotis et al., 1999). Subsequent studies confirmed that VM is involved in the growth and metastasis of solid tumors (Hendrix et al., 2003). Moreover, anti-angiogenesis alone could promote the VM formation (van der Schaft et al., 2004). Therefore, anti-VM is expected to become another effective treatment, particularly in combination with other classic means in HCC.

Erythropoietin-producing hepatocyte receptor A2 (EphA2) has a wide range of physiological and pathological functions in human development and diseases. As an oncogenic biomarker, EphA2 is associated with the poor prognosis of a variety of malignant tumors (Ieguchi and Maru, 2019). Recent studies suggest that EphA2 is involved in tumor invasion, angiogenesis, tumor matrix degradation and tumor cell adhesion (Xiao et al., 2020). A series of studies have shown that EphA2 is associated with vascular mimicry in breast cancer, gastric cancer and prostate cancer (Li et al., 2018; Kim et al., 2019a; Kim et al., 2019b; Yeo et al., 2019; Mitra et al., 2020). However, few studies have focused on the role of EphA2 in vascular mimicry of HCC (Li et al., 2020).

Traditional Chinese medicine (TCM) has been used for the adjuvant and supportive treatment of HCC for hundreds of years in China (Shi et al., 2017). As a traditional herb, *Celastrus orbiculatus* is often used to treat cancers, arthritis and a variety of inflammatory diseases (Shen et al., 2019). Our previous researches confirmed that *Celastrus orbiculatus* ethylacetate extract (COE) can inhibit VM in HCC cells by down-regulating Notch1 (Jue et al., 2017b). However, the effect of TCM on inhibiting cancers is often accomplished through multiple pathways. Whether COE inhibits vascular mimicry of HCC through other targets than Notch1 is still unknown.

In this study, proteomics was applied to screen the differential proteins in HCC cells treated with COE, and then the uniformity between genes and proteins was identified. In the six differential proteins, EphA2 was further investigated and confirmed to be a potential target of COE in anti-VM in HCC.

## Materials and Methods

### Preparation of COE

*Celastrus orbiculatus* Thunb (Sapindus order Celastraceae, www.theplantlist.org) was planted in Guangxi Province (south-west areas of China), harvested and processed by Zhixin Pharmaceutical Company (SN: 170812, Guangzhou, China). Herb identification and extraction procedure has been described previously (Qian et al., 2012; Jiang W. et al., 2019). Briefly, COE was dissolved in dimethyl sulfoxide (DMSO) as a 0.016 g/ml stock solution. When used, the stock solution was diluted into different working concentrations using culture medium. In this study the final concentration of DMSO in working fluid was less than 0.01%.

### Chemical Analysis for COE

The COE (1.0 g) was subjected to silica gel CC, 15 fractions (Fr.1-Fr.15) were collected by eluting with a gradient system of increasing polarity from PE-EtOAc (100:0–2:1) to CH_2_Cl_2_-MeOH (100:0–0:100). Fr.4-Fr.13 were subsequently subjected to ODS column, eluted with MeOH-H_2_O (20:90–100:0), then further purified by HPLC using a H_2_O/MeOH linear gradient to obtain pure compounds. Their structures were elucidated by interpretation of NMR and MS spectroscopic data. NMR spectra were measured on a Bruker Avance-600 spectrometer with TMS as the internal standard. HRESIMS spectra were recorded on a maXis spectrometer and LC-MS were measured on an Agilent 1100 series LC/MSD trap ESI spectrometer. The HPLC-DAD method was used for the identification and characterization of the compounds in the COE. The analytical HPLC were performed on a Hitachi L-2130 apparatus with a Hitachi L-2455 diode array detector. COE (1 mg) was dissolved in MeOH (1 ml) and the resulting solution (1,000 ppm) was used for HPLC-DAD analysis. A reversed phase Apollo C18 (5 μm, 4.6 mm × 250 mm) column was employed for the analysis of the COE and all the isolated pure compounds. The mobile phase was consisted of HPLC grade acetonitrile (A) and water (B), using a gradient elution of A:B (v/v): 45:55 hold for 2 min, 45:55–65:35 over 23 min, 65:35–80:20 over 2 min, 80:20–90:10 over 3 min, 90:10–95:5 over 20 min, 95:5 hold for 5 min. The flow rate was 1.0 ml/min, and 20 µl of sample was injected. The detector wavelength was set at 195 nm. The total run time was 55 min.

### Cell Culture

Human hepatocellular carcinoma cell lines MHCC97-H and HepG2 were purchased from Zhong Qiao Xin Zhou Biotech Company (Shanghai, China). Cells were maintained in Dulbecco's Modified Eagle Medium (DMEM, GIBCO, Grand Island, NY, United States) containing 10% fetal bovine serum (Hyclone, Logan, United States), 100 U/ml penicillin, 100 mg/ml streptomycin, and 2 mmol/l L-glutamine, and cultured at 37°C in an incubator with 95% humidity and 5% CO_2_ condition.

### Cell Invasion Assay

Cell invasion assay were processed as previous description (Li et al., 2019). Briefly, 24-well transwell units with polycarbonate filters (pore size, 8 μm) was coated with Matrigel on the upper side (Becton Dickinson Labware, Bedford, MA, United States). After solidification, 100 ml medium containing 1 × 10^3^ cells was added in the top chamber. The bottom chamber contained 10% fetal calf serum medium. After 24 h incubation, noninvasive cells were removed, migrated cells on the bottom surface of the membrane were fixed in formaldehyde, stained with 0.1% crystal violet solution, and counted under an Olympus IX51 inverted microscope (Olympus, Tokyo, Japan).

### VM Formation Observation

Experiment procedure has been described previously (Xue et al., 2020). Briefly, each well of a 24-well tissue culture plate was coated with 200 µl growth factor-reduced matrigel (BD Biosciences, Bedford, MA, United States), and solidified at 37°C for 60 min before plating. Cell suspension (1 × 10^5^ cells/well) was added on to the surface of the matrigel and incubated at 37°C for 72 h and followed by photographing with an Olympus IX51 inverted microscope (Olympus, Tokyo, Japan).

### Proteomics Analysis

#### Protein Extraction and Trypsin Digestion

MHCC97-H cells treated with and without COE were sonicated three times on ice using a high intensity ultrasonic processor (Scientz) in lysis buffer (8 M urea, 1% Protease Inhibitor Cocktail). The remaining debris was removed by centrifugation at 12,000 g at 4°C for 10 min. Finally, the supernatant was collected and the protein concentration was determined with a BCA kit according to the manufacturer’s instructions. The protein solution was reduced with 5 mM dithiothreitol for 30 min at 56°C and alkylated with 11 mM iodoacetamide for 15 min at room temperature in darkness. The protein sample was then diluted by adding 100 mM TEAB to urea concentration less than 2M. Finally, trypsin was added at 1:50 trypsin-to-protein mass ratio for the first round of digestion overnight and 1:100 trypsin-to-protein mass ratio for a second 4 h-digestion.

#### TMT/iTRAQ Labeling and HPLC Fractionation

After trypsin digestion, peptide was desalted by Strata X C18 SPE column (Phenomenex) and vacuum-dried. Peptide was reconstituted in 0.5 M TEAB and processed according to the manufacturer’s protocol for TMT kit/iTRAQ kit. Briefly, one unit of TMT/iTRAQ reagent were thawed and reconstituted in acetonitrile. The peptide mixtures were then incubated for 2 h at room temperature and pooled, desalted and dried by vacuum centrifugation. The tryptic peptides were fractionated into fractions by high pH reverse-phase HPLC using Agilent 300 Extend C18 column (5 μm particles, 4.6 mm ID, 250 mm length). Briefly, peptides were first separated with a gradient of 8–32% acetonitrile (pH 9.0) over 60 min into 60 fractions. Then, the peptides were combined into 18 fractions and dried by vacuum centrifuging.

#### LC-MS/MS Analysis

The tryptic peptides were dissolved in 0.1% formic acid (solvent A), directly loaded onto a home-made reversed-phase analytical column (15 cm length, 75 μm i.d.). The gradient was comprised of an increase concentration from 6 to 23% solvent B (0.1% formic acid in 98% acetonitrile) over 26 min, 23–35% in 8 min and climbing to 80% in 3 min then holding at 80% for the last 3 min, all at a constant flow rate of 400 nl/min on an EASY-nLC 1000 UPLC system.

The peptides were subjected to NSI source followed by tandem mass spectrometry (MS/MS) in Q Exactive™ Plus (Thermo) coupled online to the UPLC. The electrospray voltage applied was 2.0 kV. The m/z scan range was 350–1,800 for full scan, and intact peptides were detected in the Orbitrap at a resolution of 70,000. Peptides were then selected for MS/MS using NCE setting as 28 and the fragments were detected in the Orbitrap at a resolution of 17,500. A data-dependent procedure that alternated between one MS scan followed by 20 MS/MS scans with 15.0 s dynamic exclusion. Automatic gain control (AGC) was set at 5E4. Fixed first mass was set as 100 m/z.

### Protein Data Analysis

Protein data analysis was performed according to previous description (Song et al., 2020). Briefly, the raw data were converted into mgf files *via* Proteome Discoverer 1.4 (Thermo, American), the mgf data were further analyzed with Protein Pilot 5.0 (AB Sciex, United States). Database searching were processed using Paragon algorithm integrated in Protein Pilot 5.0. The custom database consisted of protein sequences predicted from RNA data. To increase confidence levels, proteins with iTRAQ ratios above 20 or below 0.05 were excluded, and only proteins with a reasonable ratio in all channels were considered quantifiable. Further functional analysis was performed for differential protein expression analysis, including whether the proteins were downregulated or upregulated. The change was determined compared to the CK, and *p* < 0.05 in the *t* test was used to indicate a significant difference between *Spica Prunellae* cultivated under salt stress and its blank control. The protein showing an average fold change of ≥1.3 or ≤0.77 in the experiment and with a minimum of two peptide matches were considered significantly differentially expressed. QuickGO software was used for Gene Ontology (GO) analysis of differentially expressed proteins (DEPs); the software searched the databases most commonly used in bioinformatics research to generate biological process, molecular function and cellular composition information for *Spica Prunellae*. The Kyoto Encyclopedia of Genes and Genomes (KEGG) database was used to exploit the current biochemical pathways and other types of molecular interactions.

### Reverse Transcription PCR

Trizol reagent (Invitrogen, San Diego, CA, United States) was used to isolate total RNA, and reverse transcription kit (PrimeScript™ Synthesis kit, Takara Bio, Inc., Dalian, China) was used to synthetize first strand of cDNA. RT-PCR was performed using the SYBR Premix Ex Taq Kit (Takara Bio, Inc., Dalian, China) on an Applied Biosystems 7500 Real Time PCR system (Applied Biosystems, White Plains, NY, United States). GAPDH was the internal control. Experiment was performed in triplicate. Data were shown as the fold changes. Primers for each target were shown in Table 1.

**TABLE 1:** The primers.

Primer	Sequence of primer (5′→3′)	amplification (bp)
h-LIX1L-F	CAA​GGG​CAA​ATC​AAT​GTT​GGA​G	133
h-LIX1L-R	CCG​GTG​CGA​ATA​ATG​AGC​CA	
h-HIST2H2BE-F	ATG​CCT​GAA​CCG​GCA​AAA​TC	287
h-HIST2H2BE-R	TGG​ATC​TCG​CGG​GAT​GTG​AT	
h-MYO1F-F	CAA​GCA​GAT​GCC​CTA​CTT​CAC	120
h-MYO1F-R	TCG​ATA​AGC​ATG​TTC​CGG​TAC​A	
h-SBF2-F	TCA​TCG​TGG​TAG​GCT​ATG​ACC	133
h-SBF2-R	CCA​GGC​TGA​CAA​AAC​AAC​TCA	
h-HOMER1-F	CCG​GGC​AAA​CAC​CGT​TTA​TG	100
h-HOMER1-R	TGC​TAG​TCG​AGC​AGC​TTC​TTT​A	
h-GAPDH-F	GGA​GCG​AGA​TCC​CTC​CAA​AAT	197
h-GAPDH-R	GGC​TGT​TGT​CAT​ACT​TCT​CAT​GG	
h-COX6A1-F	AGT​TGG​TGT​GTC​CTC​GGT​TTC	117
h-COX6A1-R	GTG​AGA​GTC​TTC​CAC​ATG​CGA	
h-EphA2-F	TGG​CTC​ACA​CAC​CCG​TAT​G	102
h-EphA2-R	GTC​GCC​AGA​CAT​CAC​GTT​G	

### Western Blot Analysis

Western blot analysis was performed as previously described (Chen Y. et al., 2020). Briefly, cell lysates were separated on 12% sodium dodecyl sulfate-polyacrylamide gel electrophoresis (SDS-PAGE) gels and then transferred onto nitrocellulose membranes. Sources and dilution of antibodies were shown in Table 2. HRP conjugate immunoglobulin was used as a secondary antibody (Jackson ImmunoResearch Laboratories, West Grove, PA, United States). West Pico chemiluminescent (Pierce) was used as the substrate to visualize protein bands, which were quantified using densitometric image analysis software (Image Master VDS; Pharmacia Biotech). Normalization was made against GAPDH.

**TABLE 2:** The antibodies.

Name	Mol. Wt (KD)	Dilution	Company	Catalogue number
VE-cadherin	87	1/1,000	abcam	ab33168
MMP2	74	1/1,000	abcam	ab97779
MMP9	92	1/1,000	abcam	ab38898
E-cadherin	130	1/1,000	proteintech	20874-1-AP
Twist1	21	1/50	abcam	ab50887
EphA2	125	1/1,000	cst	6997

### Animals and Xenograft Assay

Protocols were the same as previously published article (Wu et al., 2020). In brief, 25 male athymic BALB/c mice aged 3–6 weeks, 18–20 g, were obtained from the Comparative Medicine Center of Yangzhou University (Yangzhou, Jiangsu, China) and maintained in a laminar flow cabinet under specific pathogen-free conditions. Five mice were kept in each metal cage at 22 ± 2°C temperature, 55 ± 5% relative humidity, and 12 h light-dark cycle. Protocols were in accordance with internationally accepted guidelines on the use of laboratory animals, and approved by the Institutional Animal Care and Use Committee (IACUC) of Yangzhou University.

MHCC97-H cells were resuspended in 100 ml PBS with 1 × 106 cells/ml concentration for left armpit subcutaneous injection into nude mice. After the appearance of tumor with a 3 mm^3^ volume, all tumor-bearing mice were enrolled randomly into subgroups (5 mice per group) and COE (20, 40, and 80 mg/kg) or saline were administered through a gastric tube every day until the end of the experiment (Qian et al., 2012). During the experiment, the length (L), width (W) and height (H) of tumors were measured with a caliper; tumor mass was calculated according to the previous report (Nogueira et al., 2020).

### Immunohistochemistry Assay

Sections (4 μm) of the fixed xenografts were stained with IHC. All slides were dewaxed and rehydrated. Endogenous peroxidase activity was blocked and antigen retrieval was preformed. After nonspecific binding sites were blocked with 1% BSA, sections were incubated overnight at 4°C using EphA2 (1:50, PA5-36108, Invitrogen). Sections were incubated with appropriate secondary biotinylated goat anti-rabbit IgG. Finally, the sections were counterstained with hematoxylin followed by dehydration and coverslip mounting.

### Clinical Samples and Assay

We have set up a database with specimens for HCC patients in the previous study (Jue et al., 2017a). In this study we chose 10 samples confirmed with VM and without VM. VM was detected as above description. Expression of EphA2 in samples was detected as the previous report (Cho et al., 2020). For the immunofluorescence analysis, paraffin-embedded human HCC sections were stained with rabbit anti-EphA2 (Invitrogen) antibody, followed by FITC-conjugated anti-rabbit IgG antibody (Invitrogen). Positive cells were detected by confocal laser scanning microscopy (Nikon Eclipse TE2000-U). This study was approved by the institutional ethic committee of the Second People’s Hospital of Taizhou (NO. TZEYLL20180301, Taizhou, Jiangsu, China).

### PAS-CD31 Dual Staining

Experiment procedure was processed as previously described (Zhu et al., 2020). CD31 was stained with IHC the same as the above mentioned. After IHC staining of CD31, PAS staining was performed using a PAS staining kit (SN: DG0005, Leagene Biotechnology Co., Ltd, Beijing, China). Briefly, after DAB reaction, sections were treated with 0.5% periodic acid solution for 10 min and rinsed with distilled water for 5 min, followed by staining in Schiff solution for 20 min. Finally, sections were counterstained using hematoxylin, dehydrated, cleared and mounted.

### Statistical Analysis

Statistical analyses were performed using SPSS 13.0 for Microsoft Windows (SPSS Inc., Chicago, IL, United States). Continuous variables were expressed as the means ± SD and were compared between groups by using the Student’s *t*-test. Categorical variables were compared by using the Chi-Square test. The Mann–Whitney test was for non-normal distributive data. *p* < 0.05 was considered statistically significant.

## Results

### Informations Derived From Chemical Analysis for COE

**FIGURE 1:**
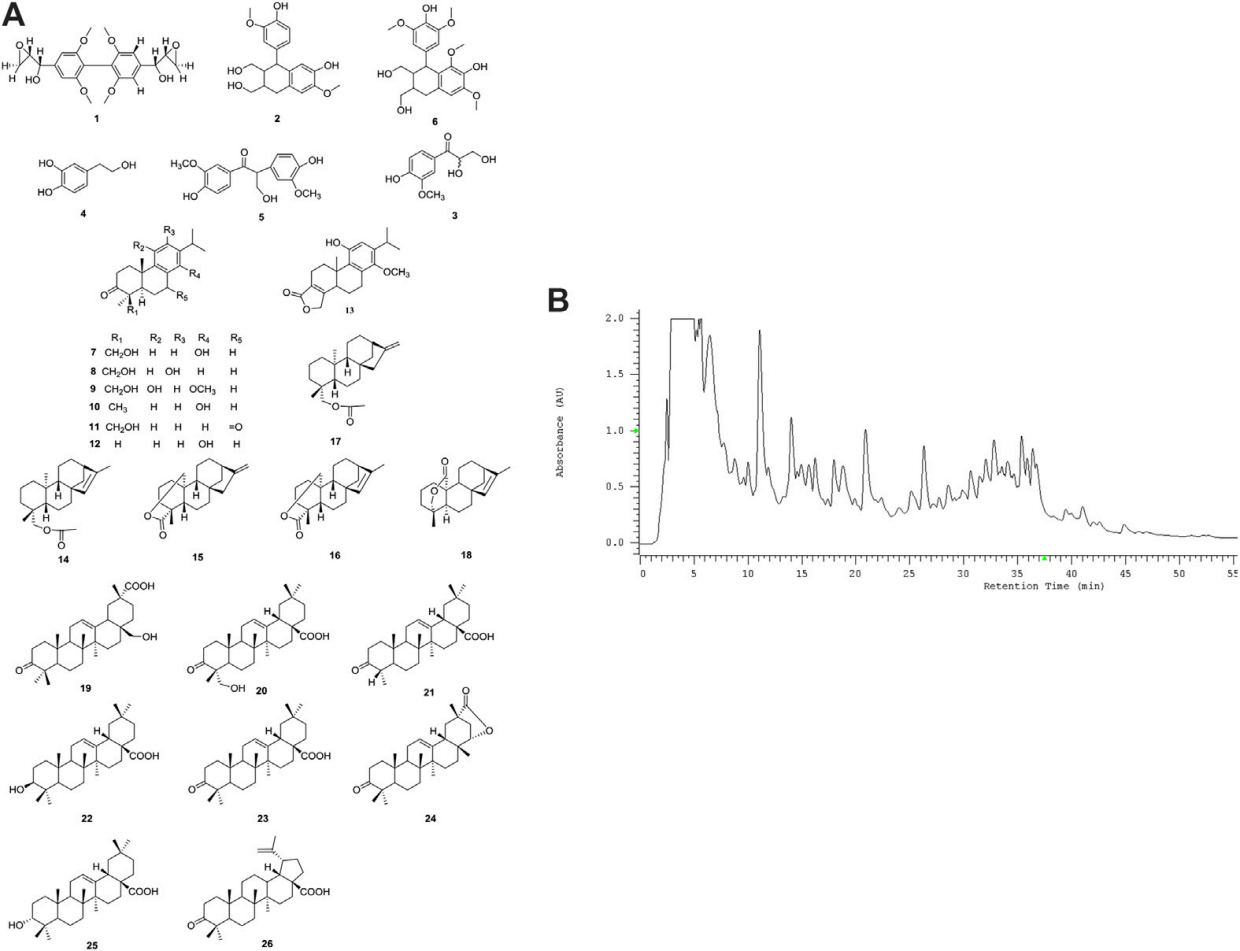
Chemical analysis for COE. **(A)** Structures and names for twenty-six compounds isolated from COE. **(B)** HPLC DAD chromatogram of COE.

Twenty-six compounds (**1**-**26**) in three different structural classes were isolated from COE (Figure 1A), including six phenylpropanoids (**1**-**6**), twelve diterpenoids (**7**-**18**) and eight triterpenoids (**19**-**26**), namely, 2,6,2′,6′-Tetramethoxy-4,4′-bis(2,3-epoxy-1-hydroxypropyl) biphenyl (**1**, 2.2 mg), (+)-isolariciresinol (**2**, 5.0 mg), 2,3-dihydroxy-1-(4-hydroxy-3-meth-oxyphenyl)-propan-1-one (**3**, 2.5 mg), 3,4-dihydroxyphenylethan-ol (**4**, 3.6 mg), Evofolin-B (**5**, 3.4 mg), (-)-lyoniresi-nol (**6**, 2.0 mg), Triptobenzene A (**7**, 3.2 mg), Triptobenzene M (**8**, 2.0 mg), Triptonediol (**9**, 4.2 mg), Triptonoterpene (**10**, 2.1 mg), Triptobenzene N (**11**, 3.8 mg), Nortriptonoterpene (**12**, 1.7 mg), Neotriptophenolide (**13**, 2.3 mg), Ent-Kaur-16-en-19-ol acetate (**14**, 4.7 mg), Ent-Kaur-16-en-19, 20-olide (**15**, 3.0 mg), Ent-Kaur-15-en-19, 20-olide (**16**, 5.6 mg), Ent-Kaur-16-en-19-ol acetate (**17**, 5.3 mg), Doianoterpene A (**18**, 5.6 mg), 28-Hydroxy-3-oxo-olean-12-en-29-oic acid (**19**, 3.0 mg), Hederagonic acid (**20,** 4.3 mg), Hedragon-ic acid (**21**, 2.5 mg), 3β-Oleanolic acid (**22**, 7.6 mg), 3-ketooleanolic acid (**23**, 1.9 mg), Wilforlide B (**24**, 2.3 mg), 3α-Oleanolic acid (**25**, 2.7 mg) and Betulonic acid (**26**, 3.4 mg).

HPLC-DAD analysis of COE (Figure 1B). Compound **3** (2.81′), **6** (2.85′), **4** (2.87′), **5** (3.22′), **2** (3.29′), **1** (*t*_R_ = 4.29′), **9** (11.05′), **7** (11.26′), **8** (11.39′), **11** (14.20′), **13** (16.50′), **12** (18.90′), **19** (21.19′), **10** (22.53′), **20** (26.56′), **18** (32.00′), **15** (32.31′), **23** (32.93′), **25** (33.30′), **21** (35.40′), **26** (35.60′), **22** (36.21′), **16** (36.47′), **17** (36.50′), **24** (36.59′), **14** (37.00′). The order of description is consistent with the appearance of peaks in the spectrum.

The preliminary cytotoxic activity of the all isolated compounds were evaluated, using MTT against human hepatoma cells line HepG2 (Jiang W. et al., 2019). The pure compounds which isolated from the COE showed much less active than the COE. It suggested that the combination of diterpenoids (**7**-**18**) as well as triterpenoids (**19**-**26**) may show synergistic effect on cytotoxic activity. Considering these we performed the following observations using COE.

### COE Inhibits VM Formation Both *In Vitro* and *In Vivo*

**FIGURE 2:**
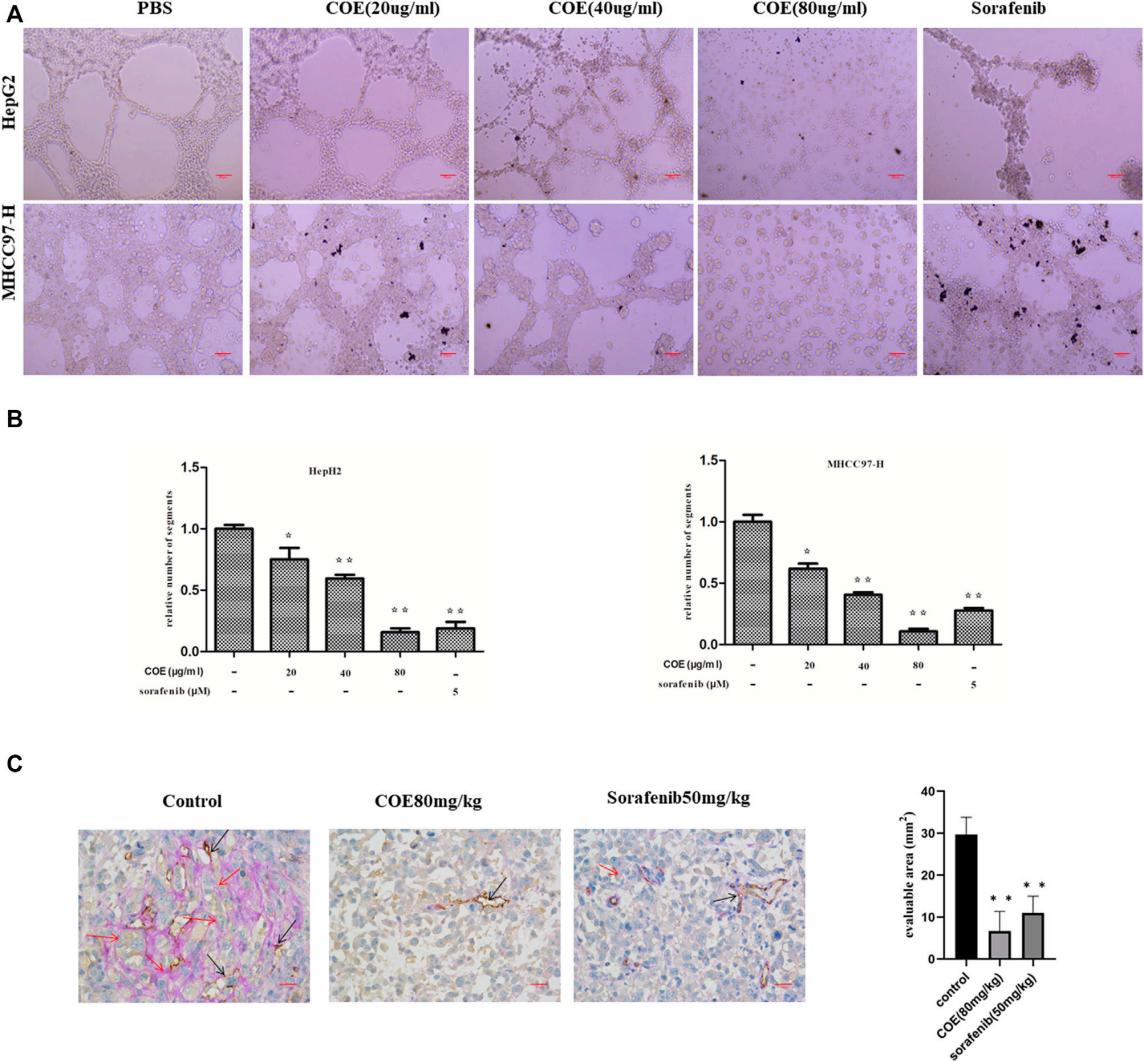
COE inhibits VM formation both *in vitro* and *in vivo*. **(A)** COE inhibits networks and loops formed by HepG2 and MHCC97-H cells on Matrigel surface ×200. Scale bar, 20 µm. **(B)**, comparison of networks among negative control, COE (20, 40, and 80 μg/ml) treated cells and positive control (sorafenib 5 µM) treated cells. Relative numbers derived from Image J. Two-tailed *t*-test. Error bars show s.e.m. ∗, *p* < 0.05, ∗∗, *p* < 0.01, vs. negative control. **(C)**, COE inhibits VM formation in MHCC97-H xenograft. Left: CD31-PAS staining in MHCC97-H tumors. Right: statistics for VM vessels, number indicates quantity of VM vessels per mm^2^. ×400, scale bar, 50 µm, ∗∗, *p* < 0.01, versus negative control.

To observe the effect of COE on VM formation in HCC cells, two HCC cell lines, HepG2 and MHCC97-H were used. When seeded on a Matrigel surface, HepG2 and MHCC97-H cells formed loops and networks in control groups. After treated with indicated concentrations of COE, networks formed by HepG2 and MHCC97-H cells gradually reduced and finally disappeared in cells treated with high concentration of COE. Sorafenib, a polyenzyme inhibitor, was reported promising in HCC treatment, thereby it was used as a positive control. Interestingly, 5 µM sorafenib effectively disrupted VM formation (Figures 2A,B). To evaluate the effect of COE in inhibiting VM *in vivo*, a HCC tumor xenograft mouse model was established by subcutaneous injection of MHCC97-H cells into nude mice. Six days post-implantation, COE and sorafenib were administrated orally once daily from the 7th day until the end of the experiment. The xenografts were dissected and examined for VM structure by double staining for CD31 and periodic acid-Schiff (PAS). Tumors derived from the negative control exhibited more VM than those from COE and sorafenib treated groups (Figure 2C). Collectively, the results from both *in vivo* and *in vitro* studies suggested that COE can effectively inhibit VM formation.

### Proteomics Analysis Indicates Differential Proteins Targeted by COE

**FIGURE 3:**
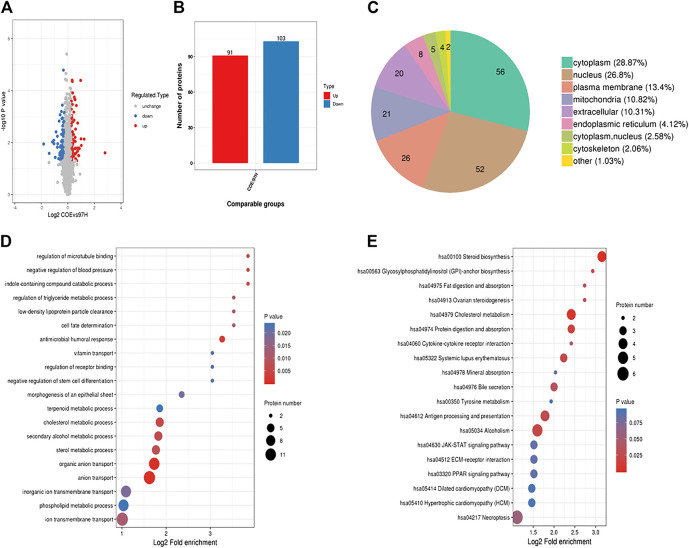
Proteomics analysis on differential proteins in COE treated MHCC97-H cells. **(A)**, volcano-plots for differential proteins. Blue dots represent down-regulated and red dots represent up-regulated proteins, black indicates no change. **(B)**, histogram for the number of significantly changed proteins. **(C)**, pie graph for origination of differential proteins in MHCC97-H cells. **(D)**, GO analysis for related proteins. **(E),** KEGG analysis for related proteins.

We next used proteomics analysis to screen the potential targets of COE. After treating MHCC97-H cells with COE (80 µg/ml) for 24 h, the total protein of cells was extracted for analysis. A total of 194 differential proteins were found via protein mass spectrometry (Figure 3A), among them 91 were significantly up-regulated and 103 down-regulated (Figure 3B). In the differential proteins 28.87% (56/194) was from cytoplasm, 26.8% (52/194) from nucleus and 13.4% (26/194) from plasma membrane (Figure 3C). Furthermore, functions of interested proteins were enriched and analyzed *via* gene ontology (GO) and KEGG database (Figures 3D,E). From the enrichment analysis, some significantly altered proteins were found to be associated with cell physiological and pathological functions. These results revealed that, the potential targets of COE, one or more of them, could disrupt multiple functions of HCC cells.

### Evaluating the Interested Targets Derived From Proteomics Analysis

**FIGURE 4:**
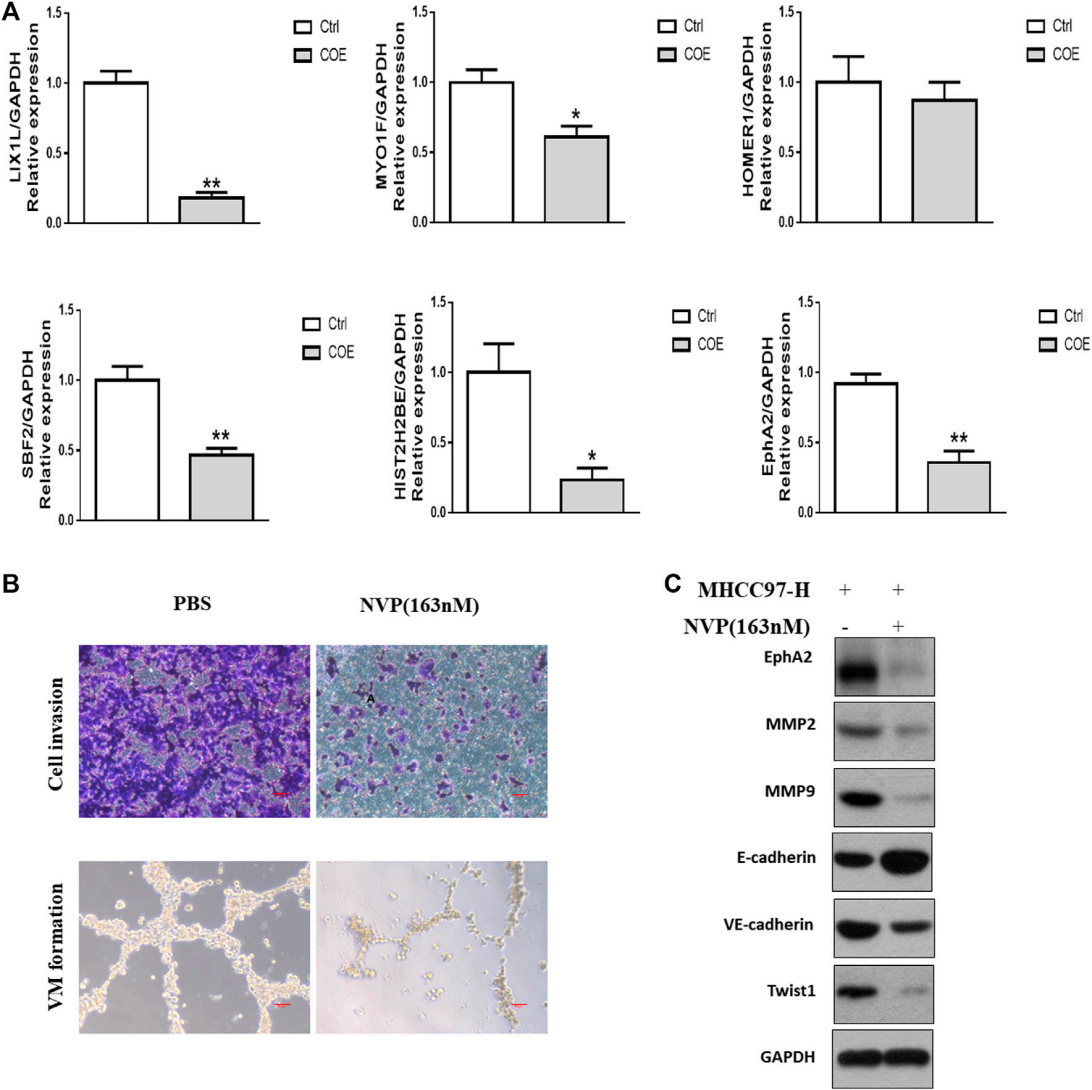
Evaluation of targets derived from proteomics analysis. **(A),** RT-PCR assay for the most significantly down-regulated genes. ∗, *p* < 0.05, ∗∗, *p* < 0.01, versus negative control. Ctrl, negative control (PBS), Two-tailed *t*-test. **(B)**, upper panel, cell invasion assay, scale bar, 20 μm lower panel, VM formation assay, scale bar, 50 µm NVP, NVP-BHG712. **(C)**, Western blotting analysis on the expression of VM related protein biomarkers after blocking EphA2.

Among the 103 down-regulated proteins, we focused on the six most decreased proteins and verified the expression of their mRNAs. As shown in Figure 4A, expressions of EphA2 and other 5 genes were significantly decreased on mRNA level after treated with COE (80 μg/ml). The tendency was in consistent with the proteomics analysis. According to published reports, EphA2 was involved in angiogenesis and VM formation (Kim et al., 2019a; Incerti et al., 2020). We further evaluated VM formation in MHCC97-H cells treated with NVP-BHG712, a tyrosine kinase receptor inhibitor (TKI) which could selectively bind to EphA2 and block its signal (Troster et al., 2018). As shown in Figure 4B, NVP-BHG712 at 163 nM concentration significantly decrease cell invasion, VM structure and protein biomarkers associated with VM (Figure 4C).

**FIGURE 5:**
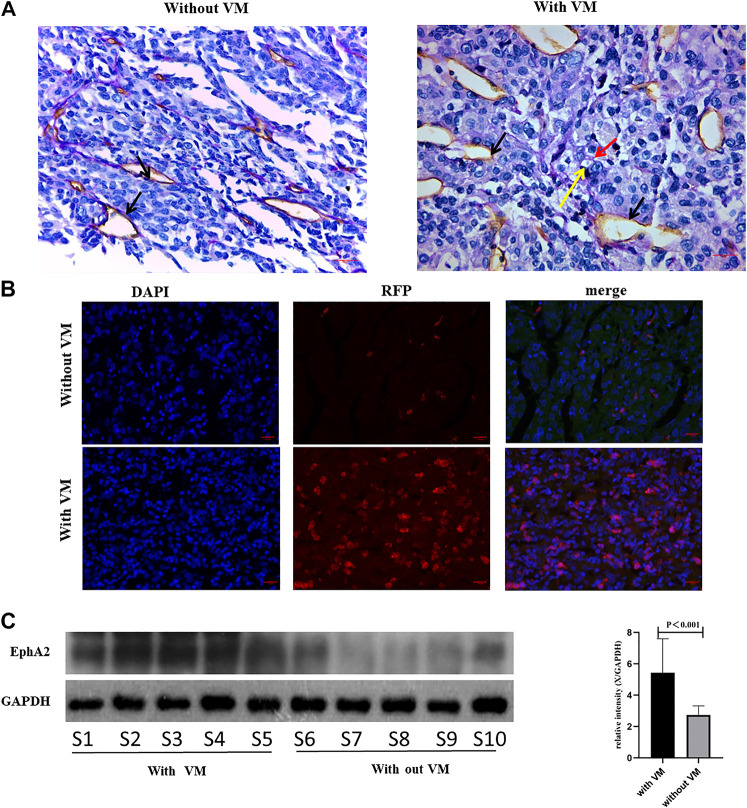
Analyzing EphA2 expression in HCC resection samples. **(A)**, CD31-PAS staining in HCC samples, red arrow indicates VM, black arrow indicates endothelial dependent vessel, yellow arrow indicates red blood cells. ×400, scale bar, 50 µm. **(B)**, IF for EphA2 expression in resection samples. DAPI, 4',6-diamidino-2-phenylindole, RFP, red fluorescent protein. ×400, scale bar, 50 µm. **(C)**, Western blot analysis on EphA2 in 10 HCC resection samples. S1 represents sample 1. Right: relative density derived from Image J process. GAPDH as the internal reference. Two tailed *t*-test.

Since blocking EphA2 resulted in damaging VM formation in HCC cells, we further examine the association between VM and EphA2 expression in clinical resection samples. Immunohistochemical and immunofluorescent staining confirmed that the tumor tissues with VM structures exhibited higher EphA2 expression than that in those without VM (Figure 5). These data indicated that EphA2 may be an important switch in VM formation and a potential target of COE in inhibiting VM formation in HCC.

### COE Inhibits Invasion and VM Associated Protein Biomarkers *via* EphA2 in HCC Cells

**FIGURE 6:**
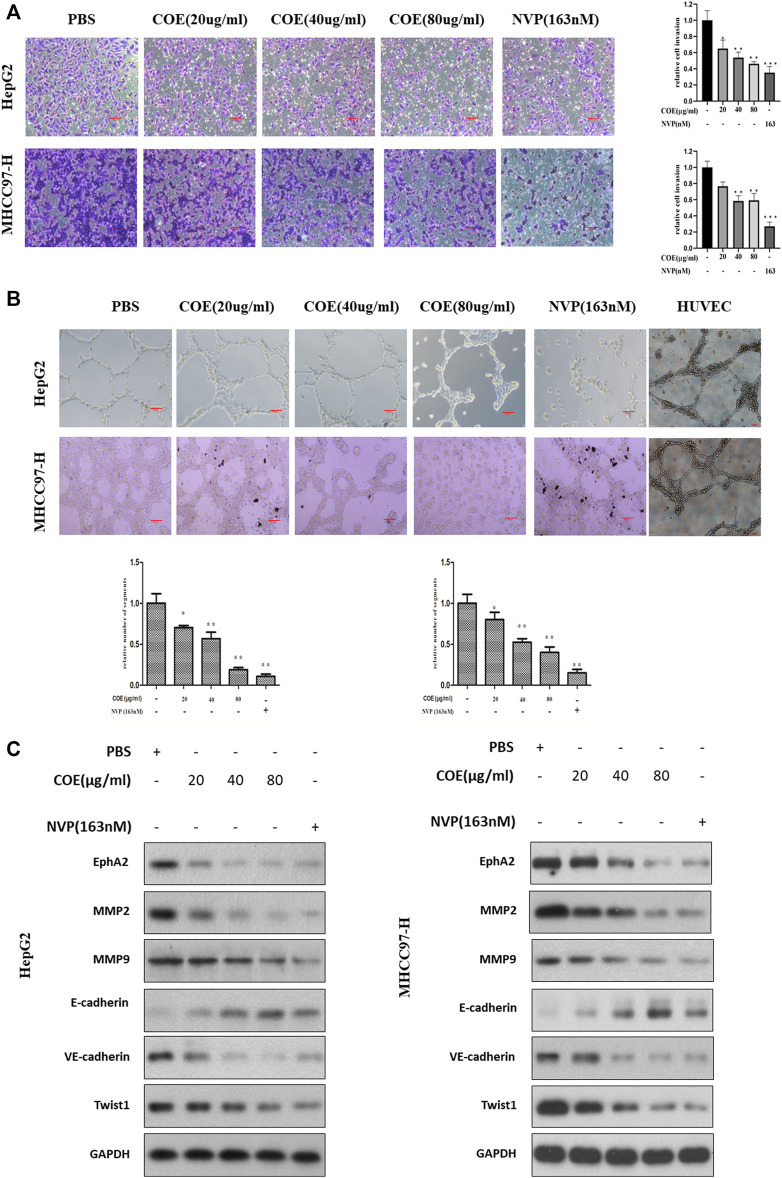
COE inhibits invasion and VM associated protein biomarkers *via* inhibiting EphA2 in HCC cells. **(A)**, COE decreases HepG2 and MHCC97-H cell invasion. **Left:** representative image for transwell assay, **right:** histogram of invaded cells. ×200, 20 µm. **(B)**, COE and NVP inhibit VM formation of HepG2 and MHCC97-H on matrigel. HUVECs used as the control. **(C)**, Western blot analysis on the change of expression of VM related proteins after COE treatment.

High invasiveness is a prominent characteristic of cancer cells with VM ability (Hess et al., 2001). Based on the above findings, we next observed the effect of COE with gradient concentrations on cell invasion in HepG2 and MHCC97-H cells with NVP-BHG712 as a positive control. As shown in Figure 6A, NVP-BHG712 decreases cell invasion, the same tendency was observed in COE treated groups. As the expression of EphA2 was inhibited by COE or NVP-BHG712, protein biomarkers related to VM formation were attenuated or enhanced accordingly (Figure 6B). Collectively, these results suggest that COE might inhibit VM formation via inhibiting EphA2 in HCC cells.

### COE Inhibits VM Formation by Inhibiting EphA2 *In Vivo*

**FIGURE 7:**
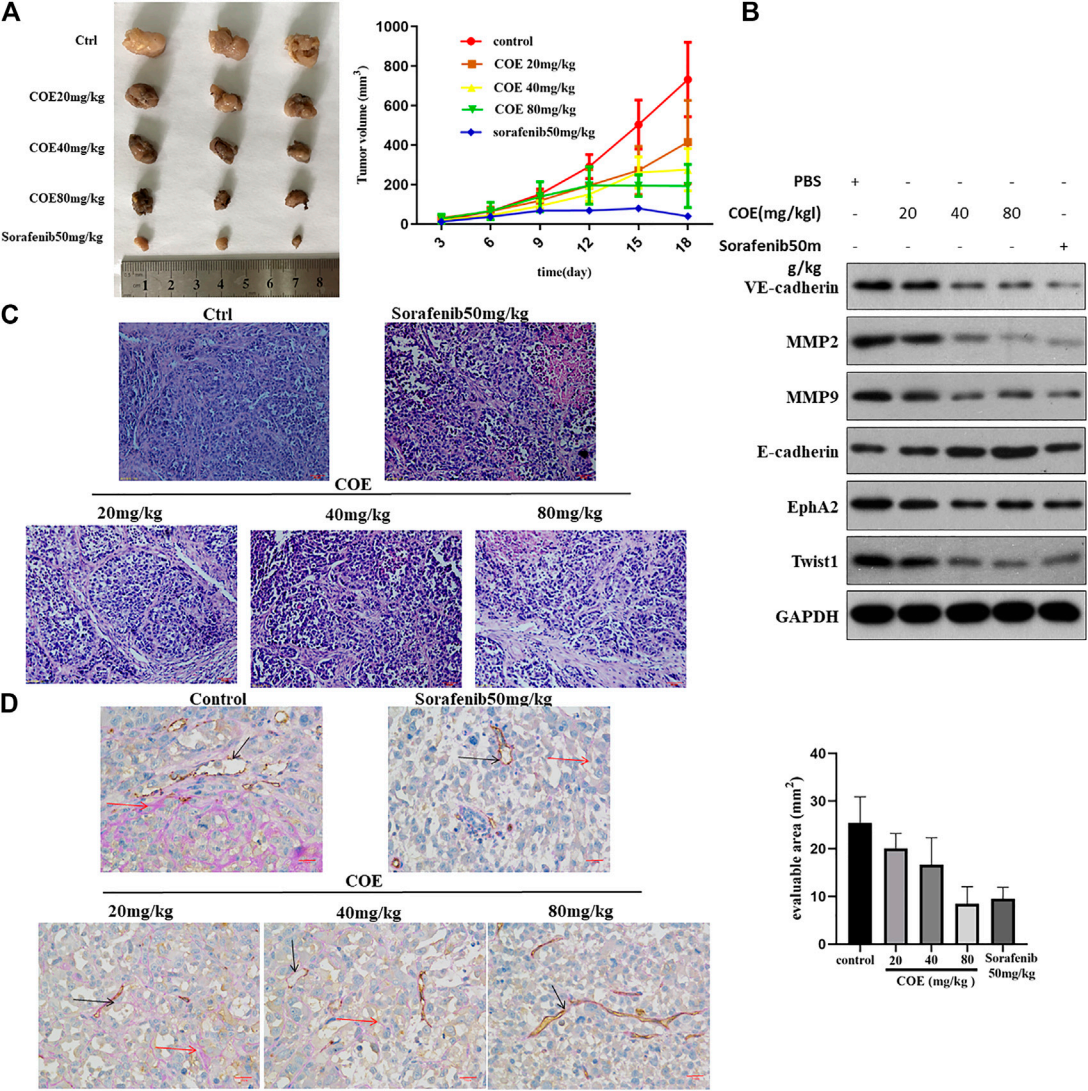
COE inhibits VM formation via inhibiting EphA2 *in vivo.* **(A)**, **left panel** indicates tumor volume in each group. **Right panel**, the curve indicates dynamic changes of tumor volume during the experiment. **(B)**, Western blotting on the expression of VM related proteins in tumor tissues. **(C)**, H&E staining on tumors in each group, ×400, 50 µm. **(D)**, left: CD31-PAS staining for VM in tumors. Right: the number of VM in evaluable area. ×400, 50 µm.

We further evaluate the effect of COE in HCC tumor xenograft *in vivo*. As shown in Figure 7A, COE decreases tumor size and volume in a concentration-dependent manner. H&E staining showed prominent cell necrosis and apoptosis in COE and sorafenib treated tumors (Figure 7C). CD31-PAS staining shows disruption of VM formation in xenograft tumors. Western blotting analysis shows the changes of expression of VM related biomarkers (Figures 7B,D). These findings suggest that COE can inhibit xenografts via inhibiting EphA2 and VM associated protein biomarkers *in vivo.* These results is in consistent with our previous finding that COE can inhibit HCC xenografts *in vivo* (Jue et al., 2017b).

## Discussion

More and more studies have demonstrated that Chinese herbs can inhibit cancer progression via anti-angiogenesis and anti-VM (Liu et al., 2015; Hou et al., 2016; Shi et al., 2016; Xu et al., 2019; Zhang et al., 2020; Zong et al., 2020). In this study, we showed that COE can inhibit VM formation of HCC cells both *in vivo* and *in vitro*. Proteomic analysis identified differential proteins and related signal pathways of HCC cells treated with or without COE. Our results suggested that COE could inhibit VM formation by down-regulating EphA2.

Our previous studies indicated that COE can inhibit VM formation of HCC by down-regulating Notch1 signaling. In this study, we confirm once again that COE can inhibit VM formation in a concentration dependent manner. In essence, VM comes from tumor cells when they acquire the functions of vascular endothelial cells (Seftor et al., 2002; Li et al., 2020). Epithelial to mesenchymal transition (ECM) related pathways and angiogenesis related pathways play an important role in this process (Shuai et al., 2020). Therefore, many signaling pathways are involved in the formation of vascular mimicry (Guan et al., 2016; Cai et al., 2020). Based on this understanding, we believe that Notch1 signaling is only one of the important targets in the process of VM in HCC cells. Therefore, the inhibition of malignant tumors should be a combination of multiple targets and multiple signaling pathways (Jiang et al., 2016). Our previous studies suggested that COE, can regulate tumor apoptosis, ECM and angiogenesis through a variety of signaling targets (Gu et al., 2016; Qian et al., 2018; Qian et al., 2019a; Qian et al., 2019b). We speculated that there may be many other potential targets for COE in inhibiting VM in HCC besides Notch1. The proteomic analysis in this study showed that nearly 200 proteins were differentially expressed in MHCC97-H cells after COE treatment. These results suggest that the inhibition of COE on VM of HCC may be achieved through the regulation of multiple targets and multiple signaling pathways.

EphA2 belongs to the family of tyrosine kinase receptor, it is activated by canonical and noncanonical pathways to promote progression of various malignant tumors (Chen Y. H. et al., 2020). Recently, EphA2 has been reported to be involved in VM development of many solid tumors (Margaryan et al., 2009; Kim et al., 2019a; Incerti et al., 2020). For example, in glioma, EphA2 overexpression is closely related to VM, and the inhibition of VM can be achieved through the regulation of EphA2 by microRNA 141 (Li et al., 2018). In gastric cancer, activation of EphA2 can trigger the downstream signaling pathways PI3K and FAK, following the cascade reactions, VM formation is promoted. Hence, EphA2 play an important role in VM of gastric cancer cells (Kim et al., 2019a). Although EphA2 was first isolated and identified from HCC cells, its specific roles and mechanisms in VM of HCC remain unclear (Li et al., 2020). In this study, our proteomic results suggest that the transcription and expression of EphA2 in HCC cells are significantly decreased after COE treatment. In addition, the invasion and VM forming ability of HCC cells are significantly inhibited by NVP, a specific inhibitor of EphA2 signaling. Following blocking of EphA2, some protein biomarkers related to VM, such as VE-cadherin, E-cadherin and twist, are changed. These results suggest that EphA2 plays a key role in the VM formation of HCC and may be another key target of COE.

Epithelial to mesenchymal transformation (EMT) and extracellular matrix (ECM) remolding are two key steps contributing to VM (Shuai et al., 2020). Matrix metalloproteinases (MMPs) are important components of the extracellular matrix proteasome, which are capable of degrading the basement membrane and type IV collagen, a key component of ECM, providing an access for cancer cells invasion and metastasis (Jiang K. et al., 2019). In this study, blocking EphA2 with COE or NVP could decrease MMP-2 and MMP-9 and inhibit VM formation, the relevant mechanism may be associated with ECM remolding. We also found the changes of E-cadherin, **VE**-cadherin and **Twist1 after COE or NVP treatment**. E-cadherin mediates the interconnection of allogeneic cells and is a member of a family of calcium-dependent adhesion molecules that mediate cell-to-cell adhesion to maintain proper morphology and polarity of tissues and participate in intracellular signal transduction (Hong et al., 2019). When E-cadherin expression is reduced or absent, intercellular adhesion will be ceased (Gloushankova, 2008). VE-cadherin belongs to the calcium-dependent adhesin family and is mainly expressed at the adhesion sites of endothelial cells. It plays an important role in endothelial cell migration and survival, angiogenesis, and maintenance of vascular integrity (Rezaei et al., 2020). Studies have found that VE-cadherin enhances the VM ability of liver cancer cells (Shuai et al., 2020). Twist1 gene is an important regulator of EMT and is generally regarded as a transit-related gene. Under the regulation of Twist1 gene, epithelial cells lose the connection with basement membrane and other epithelial phenotypes, mediating the invasion and metastasis of malignant tumor cells (Yeo et al., 2020). At the same time, studies have shown that Twist1 promoter can bind with VE-cadherin promoter after transcriptional activation, thus promoting VM in HCC cells (Xiao et al., 2018). In our previous study it has been demonstrated that COE inhibits the EMT of HCC cells (Jue et al., 2017a). Here it is parallel for improving the expression level of E-cadherin and reducing VE-Cadherin, Twist1, MMP2 and MMP9. Collectively, it is rationally considered that COE inhibits VM formation in HCC by intervening EMT.

Taken together, this study elucidates that COE can down regulate the expression of EphA2 in HCC cells and exert its inhibitory effect on HCC VM both *in vitro* and *in vivo*. Our previous studies have confirmed that COE can inhibit the VM formation of HCC by down regulating Notch1, however, is there any crosstalk between EphA2 and Notch1? This is not reflected in this study thereby is the limitation of this study. It seems that hypoxia inducible factor (HIF) plays a role as a link between the two signaling pathways, but the specific mechanism needs further examinations. In addition, the exact mechanism of EphA2 in VM formation of HCC is still unclear, which needs further research.

## Conclusion

In this study, using proteomics analysis, we identify some significantly differential proteins associated with COE treatment. Among these differential proteins, EphA2 stands out to be a key target of COE in inhibiting VM formation in HCC. The findings support that COE could be an effective drug against VM in HCC.

## Data Availability

The datasets presented in this study can be found in online repositories. The name of the repository and accession number can be found below: ProteomeXchange Consortium via the PRIDE [1] partner repository, http://www.proteomexchange.org/, PXD022203.
