# Corrigendum: COE Inhibits Vasculogenic Mimicry by Targeting EphA2 in Hepatocellular Carcinoma, a Research Based on Proteomics Analysis

**DOI:** 10.3389/fphar.2021.831941

**Published:** 2022-01-05

**Authors:** Zewen Chu, Xin Shi, Gaoyang Chen, Xuejun He, Yayun Qian, Haibo Wang, Li Tao, Yanqing Liu, Wei Jiang, Jue Chen

**Affiliations:** ^1^ Institution of Integrated Traditional Chinese and Western Medicine, Medical College, Yangzhou University, Yangzhou, China; ^2^ Department of Oncology, The Second People’s Hospital of Taizhou Affiliated to Medical College of Yangzhou University, Yangzhou, China; ^3^ College of Environmental Science and Engineering, Marine Science and Technology Institute, Yangzhou University, Yangzhou, China

**Keywords:** vasculogenesis mimicry, hepatocel lular carcinoma, EphA2, protemics, cancer treatment

In the original article, there was a mistake in Figure 2, Figure 4, and Figure 6 as published. The mistake was induced by using PPT software to import all experimental pictures of each group of drugs at one time during picture sorting. In the subsequent ranking of representative pictures, the pictures of individual concentrations were mixed with those of other concentrations. The corrected Figure 2, Figure 4, and Figure 6 appear below.

**FIGURE 2:**
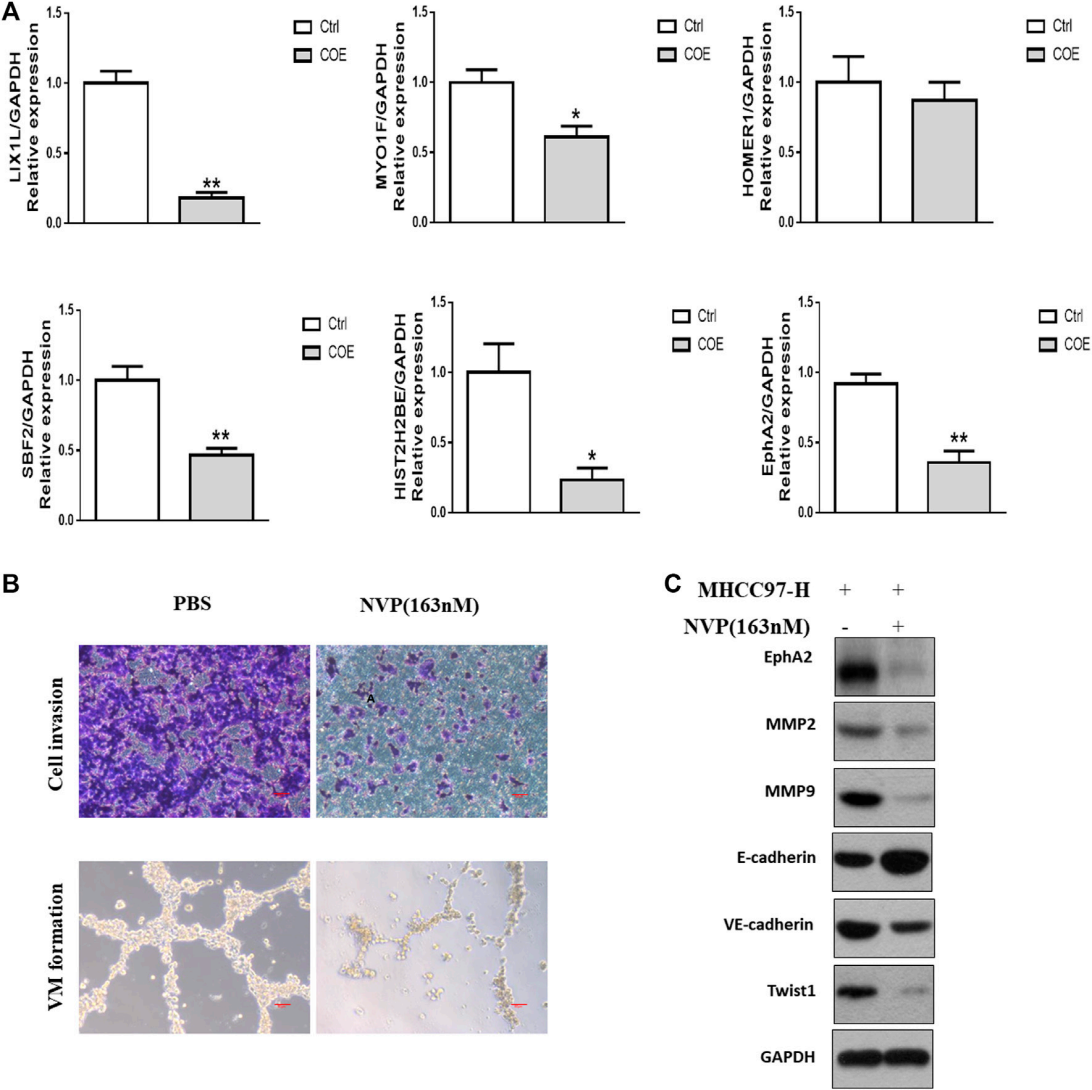
COE inhibits VM formation both *in vitro* and *in vivo*. **(A)** COE inhibits networks and loops formed by HepG2 and MHCC97-H cells on Matrigel surface ×200. Scale bar, 20 µm. **(B)**, comparison of networks among negative control, COE (20, 40, and 80 μg/ml) treated cells and positive control (sorafenib 5 µM) treated cells. Relative numbers derived from Image J. Two-tailed *t*-test. Error bars show s.e.m. ∗, *p* < 0.05, ∗∗, *p* < 0.01, vs. negative control. **(C)**, COE inhibits VM formation in MHCC97-H xenograft. Left: CD31-PAS staining in MHCC97-H tumors. Right: statistics for VM vessels, number indicates quantity of VM vessels per mm^2^. ×400, scale bar, 50 µm, ∗∗, *p* < 0.01, versus negative control.

**FIGURE 4:**
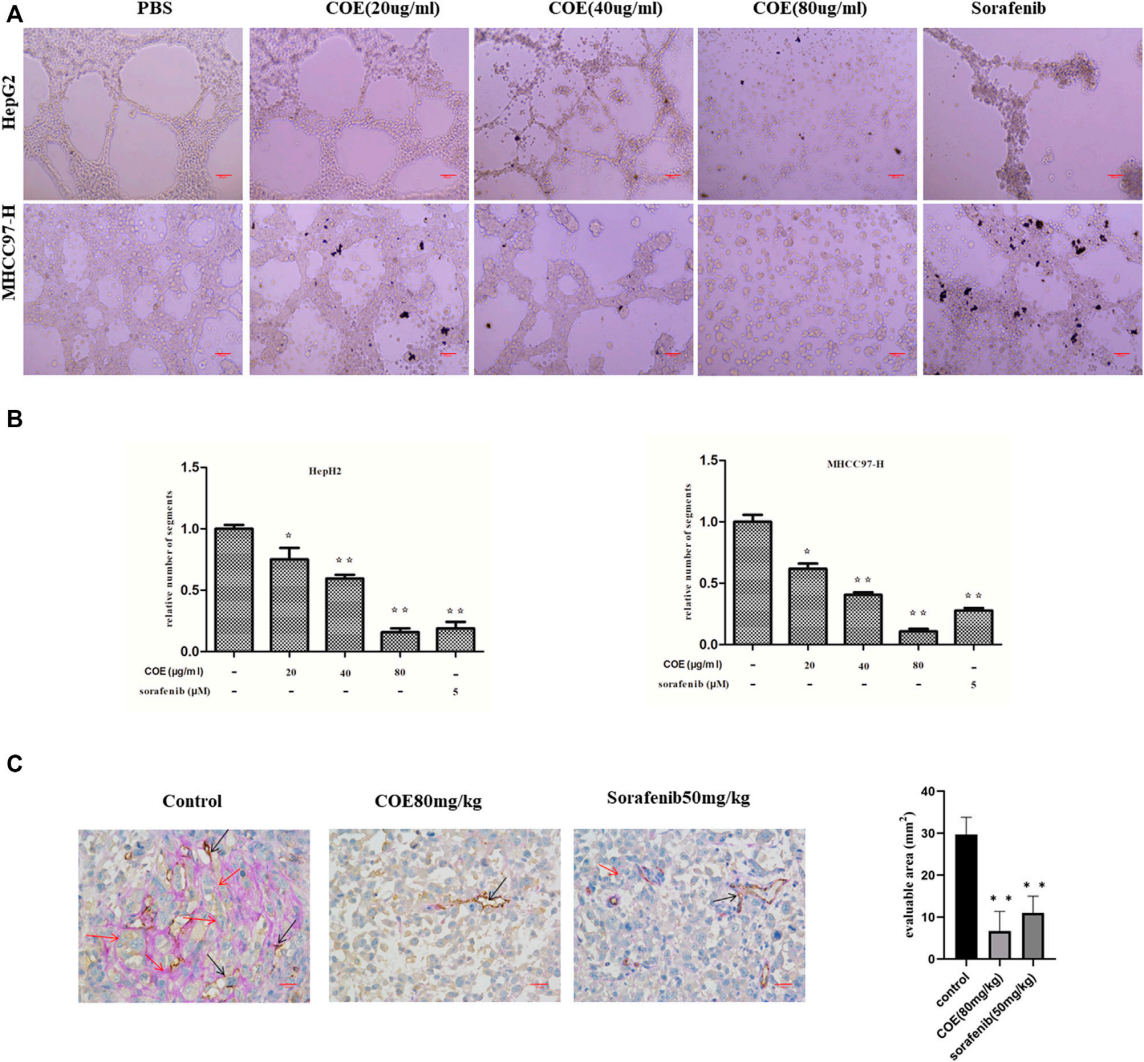
Evaluation of targets derived from proteomics analysis. **(A),** RT-PCR assay for the most significantly down-regulated genes. ∗, *p* < 0.05, ∗∗, *p* < 0.01, versus negative control. Ctrl, negative control (PBS), Two-tailed *t*-test. **(B)**, upper panel, cell invasion assay, scale bar, 20 μm lower panel, VM formation assay, scale bar, 50 µm NVP, NVP-BHG712. **(C)**, Western blotting analysis on the expression of VM related protein biomarkers after blocking EphA2.

**FIGURE 6:**
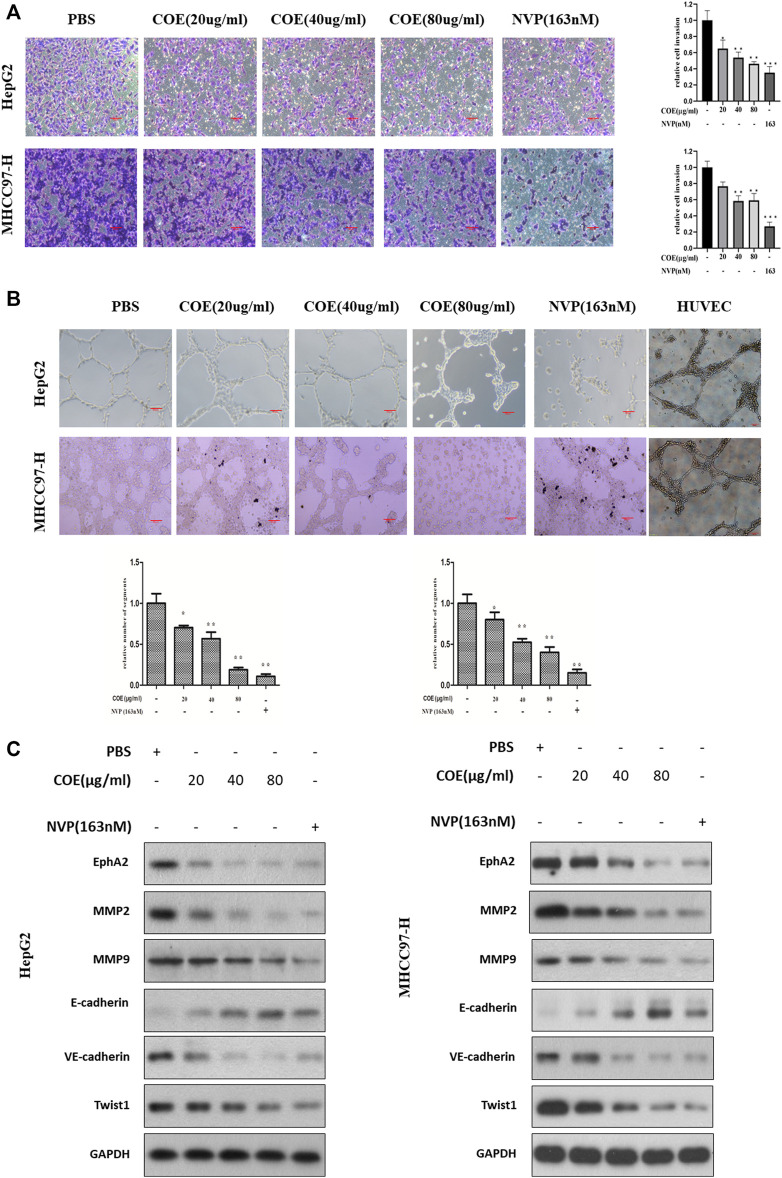
COE inhibits invasion and VM associated protein biomarkers *via* inhibiting EphA2 in HCC cells. **(A)**, COE decreases HepG2 and MHCC97-H cell invasion. Left: representative image for transwell assay, right: histogram of invaded cells. ×200, 20 µm. **(B)**, COE and NVP inhibit VM formation of HepG2 and MHCC97-H on matrigel. HUVECs used as the control. **(C)**, Western blot analysis on the change of expression of VM related proteins after COE treatment.

The authors apologize for this error and state that this does not change the scientific conclusions of the article in any way. The original article has been updated.

